# Visualization of Content Release from Cell Surface-Attached Single HIV-1 Particles Carrying an Extra-Viral Fluorescent pH-Sensor

**DOI:** 10.1371/journal.pone.0148944

**Published:** 2016-02-10

**Authors:** Chetan Sood, Mariana Marin, Caleb S. Mason, Gregory B. Melikyan

**Affiliations:** 1 Division of Pediatric Infectious Diseases, Emory University School of Medicine, Atlanta, Georgia, United States of America; 2 Children’s Healthcare of Atlanta, Atlanta, Georgia, United States of America; University of Missouri, UNITED STATES

## Abstract

HIV-1 fusion leading to productive entry has long been thought to occur at the plasma membrane. However, our previous single virus imaging data imply that, after Env engagement of CD4 and coreceptors at the cell surface, the virus enters into and fuses with intracellular compartments. We were unable to reliably detect viral fusion at the plasma membrane. Here, we implement a novel virus labeling strategy that biases towards detection of virus fusion that occurs in a pH-neutral environment—at the plasma membrane or, possibly, in early pH-neutral vesicles. Virus particles are co-labeled with an intra-viral content marker, which is released upon fusion, and an extra-viral pH sensor consisting of ecliptic pHluorin fused to the transmembrane domain of ICAM-1. This sensor fully quenches upon virus trafficking to a mildly acidic compartment, thus precluding subsequent detection of viral content release. As an interesting secondary observation, the incorporation of the pH-sensor revealed that HIV-1 particles occasionally shuttle between neutral and acidic compartments in target cells expressing CD4, suggesting a small fraction of viral particles is recycled to the plasma membrane and re-internalized. By imaging viruses bound to living cells, we found that HIV-1 content release in neutral-pH environment was a rare event (~0.4% particles). Surprisingly, viral content release was not significantly reduced by fusion inhibitors, implying that content release was due to spontaneous formation of viral membrane defects occurring at the cell surface. We did not measure a significant occurrence of HIV-1 fusion at neutral pH above this defect-mediated background loss of content, suggesting that the pH sensor may destabilize the membrane of the HIV-1 pseudovirus and, thus, preclude reliable detection of single virus fusion events at neutral pH.

## Introduction

HIV-1 fusion with a host cell is initiated after the viral Env glycoprotein forms ternary complexes with the receptor (CD4) and coreceptors (CCR5 or CXCR4) on the cell surface. The resulting refolding of the transmembrane gp41 subunit of Env into the stable six-helix bundle structure mediates merger of viral and cell membranes and release of the genetic material into the cytosol (reviewed in [1, 2]). Key interactions that are required for HIV-1 fusion, including the initial conformational changes in gp41, occur at the cell surface [3–5], whereas cellular sites of viral fusion remain controversial [6]. HIV-1 has long been thought to fuse directly with the plasma membrane. Evidence supporting this entry pathway include: (i) the formation of ternary complexes with CD4 and coreceptors on the cell surface [3, 5, 7, 8]; (ii) pH-independence of Env-mediated membrane fusion [9, 10]; and (iii) the ability of cell-expressed Env or viruses adhered to adjacent cells to promote cell-cell fusion [11–14]. However, inhibition of HIV-1 fusion/infection upon blocking virus uptake [3, 4, 15, 16] and enhancement of fusion/infection upon blocking endosomal acidification (and thus sparing the virus from degradation in lysosomes) [17–19] suggest that a large fraction of HIV-1 enters through endocytosis. Endosomal entry is supported by the observation that HIV-1 becomes resistant to fusion inhibitors that act only on virions at the cell surface earlier than to a low-temperature block that abrogates fusion irrespective of virus location [4]. Finally, single HIV-1 imaging in live cells revealed viral content release into the cytoplasm from within endosomes, but not from the cell surface [4, 20].

Discrepant findings regarding HIV-1 entry pathways in relevant target cells, macrophages and CD4 T-cells have been reported, suggesting that the site of HIV-1 fusion is cell type-dependent [4, 9, 21–25]. A major source of discordant results is the reliance on indirect assays monitoring bulk virus uptake, on one hand, and population-based functional readouts, such as viral fusion or infection, on the other [26]. Imaging single HIV-1 entry and fusion in live cells provides a powerful means to pinpoint the virus entry sites [3, 4, 27, 28].

We have previously imaged single HIV-1 fusion to determine the site of virus entry by incorporating a lipid dye into the viral membrane and trapping a releasable content marker inside the virus [3, 4]. With this labeling strategy, the disappearance of the lipid dye at the time of viral content release indicates an infinite dilution of the lipid dye to the plasma membrane and, thus, fusion at the cell surface. Retention of the membrane marker at content release sites implies a limited dilution of the lipid dye by fusion with an endosome. We have examined fusion of HIV-1 pseudoviruses with target cells, using this strategy, and concluded that this virus overwhelmingly fuses with endosomes [3, 4]. However, the above virus labeling strategy is not optimal for detecting single virus fusion with the plasma membrane, as it results in the loss (sequential or simultaneous) of both viral markers. Although rare double-disappearance events were observed [4], once a lipid dye was lost, the site of subsequent viral fusion could not be reliably deduced.

In light of limitations of the above HIV-1 labeling strategy, alternative virus labeling and imaging approaches are needed to elucidate the preferred sites of entry into different cell types and under different conditions. Here, we introduce a simple labeling strategy that allows detection of HIV-1 fusion with the plasma membrane or early pH-neutral endosomes. This was accomplished by anchoring a pH-sensitive green fluorescent protein to the exterior of the viral membrane that is fully quenched at mildly acidic pH, and incorporating a pH-insensitive red fluorescent protein, which is readily releasable upon viral fusion, into the particles. HIV-1 fusion at neutral pH—either with the plasma membrane or with early pH-neutral vesicles—should be readily detectable, since it results in the instantaneous loss of a content marker, without the low pH-mediated quenching of a pH-sensor. In contrast, virus trafficking to and subsequent fusion with an acidic endosome manifests as double-loss of signals from both the pH-sensor and content marker, precluding reliable detection of fusion events. We reasoned that biasing the imaging assay against endocytic entry events should reveal rare fusion events occurring at the cell surface with no contribution from endosomal fusion events. We found that a small fraction of pseudoviruses bound to the plasma membrane lost their content marker, but, unexpectedly, the content release could not be blocked by HIV-1 fusion inhibitors. We thus were unable to detect a significant occurrence of HIV-1 fusion at neutral pH above this background of spontaneous content release.

## Materials and Methods

### Cell lines and reagents

Dulbecco’s Modified Eagle Medium (DMEM) and Hank’s Balanced Salt Solution (HBSS) were from Cellgro (Mediatech, Manassas, VA). Fetal bovine serum (FBS) and DMEM without phenol red were from Hyclone Laboratories (Logan, UT). Live Cell Imaging Buffer (LCIB) and FluoroBrite™ DMEM were from Life Technologies (Grand Island, NY). HEK293T/17 and CV-1 cells were obtained from ATCC (Manassas, VA). The CV-1/CD4/CXCR4 (CF3 clone) and CV-1/TVA950 cell lines have been described previously [3, 29]. The HeLa-derived TZM-bl cell line expressing CD4, CXCR4 and CCR5 was obtained from the NIH AIDS Research and Reference Reagent Program (donated by Drs. J.C. Kappes and X. Wu [30]). All cell lines were cultured in Dulbecco’s Modified Eagle Medium supplemented with 10% fetal bovine serum and 100U penicillin-streptomycin (Gemini Bio-Products, Sacramento, CA). Poly-L-lysine, AMD3100, and Bafilomycin A1 were from Sigma-Aldrich (St. Louis, MO). The C52L recombinant peptide was a kind gift from Dr. Min Lu (University of New Jersey).

### Plasmids and pseudovirus production

The expression vectors HXB2, pcRev, pR8ΔEnv, HIV-1-Gag-imCherryΔEnv have been described previously [4, 27]. The YFP-Vpr plasmid was a gift from Dr. T. Hope (Northwestern University). The ecliptic pHlourin-ICAM-1 (EcpH-ICAM) chimera has been described previously [31]. In brief, EcpH and ICAM-1 transmembrane fragments were cloned into a p3xFLAG CMV9 vector. Pseudovirus production has been described previously [4, 27]. Briefly, HEK 293T/17 cells were grown to 60–80% confluency in a 6-well plate and a single well was transfected with the following amounts of plasmids: 0.6 μg HXB2 Env, 0.2 μg pcRev, 0.3 μg pR8ΔEnv, 0.3 μg HIV-1-Gag-imCherryΔEnv, and either 0.6 μg EcpH-ICAM or 0.4 μg YFP-Vpr, using JetPrime transfection reagent (Polyplus-transfection, Illkirch, France). Eight hours post-transfection the media was exchanged with pre-warmed DMEM without phenol-red. Twenty-four or forty-eight hours post transfection the supernatant was collected, syringe-filtered to 0.45 μm, aliquoted, and frozen at −80°C. Virus titer was determined by the β-Gal infectivity assay, as described previously [4].

### Single-virus imaging

Immobilized particle experiments were performed on pseudoparticles diluted in ice-cold PBS^++^ and incubated on poly-L-lysine coated coverslips for 30 min at 4°C. Coverslips were washed with ice-cold PBS^++^ to remove unbound virus. To measure the response of viral EcpH-ICAM to environmental pH, bound virus was covered in room temperature LCIB with pH titrated to 6.3, 6.8, or 7.3 by addition of HCl. Spontaneous content release experiments were initiated by addition of pre-warmed LCIB. Single-viral fusion experiments were performed on cells plated on collagen-coated glass-bottom dishes (MatTek, Ashland, MA) in FluoroBrite DMEM and grown to 90% confluency. Before imaging, the cells were chilled on ice, washed with ice-cold PBS^++^ and spinoculated with freshly thawed pseudovirus (MOI of 0.005–0.01) in LCIB at 4°C for 20 min at 1500×*g*. After spinoculation, cells were washed with ice-cold PBS^++^ to remove unbound virus and virus entry was initiated by addition of pre-warmed LCIB, HBSS or other buffers, as indicated. Imaging buffers were supplemented with 2% FBS. In control experiments, LCIB was titrated to pH 7.9 with HCl or supplemented with 10 μM AMD3100 or 100 nM Bafilomycin A1. For the latter experiments, cells were pre-treated for 1 hour at 37°C with 100 nM Bafilomycin A1. Images were acquired in a single axial plane with a Personal DeltaVision imaging system (Applied Precision, GE, Pittsburgh, PA) using an UPlanFluo 40x/1.3 NA oil objective (Olympus, Tokyo, Japan) and either a GFP/Cherry or CFP/YFP/Cherry standard filter set (Chroma, Bellows Falls, VT). Two-channel fluorescence emission was recorded in series by an EM-CCD camera (Photometrics, Tucson, AZ). For viral fusion experiments a single field-of-view was imaged every 3 sec and in spontaneous content release experiments eight fields-of-view were imaged every minute. During time-lapse imaging an environmental chamber was used to maintain samples at 37°C and high humidity and the UltimateFocus module (Applied Precision, GE) was used to compensate for axial drift.

### Event annotation, curve fitting, and single-particle tracking

Single-particle color change events were annotated using the ROI manager in Image J (NIH). Sets of events were rank-ordered by time to generate kinetic curves. Annotated particles were tracked in Volocity (Perkin Elmer, Waltham, MA) using a custom script that associated at each time point the tracked particle with a one- or two-pixel dilation of the tracked particle. A script in Excel (Microsoft, Redmond, WA) was designed to collate the tracking data and combine the tracked particle and the dilated object to find a halo surrounding the tracked particle which was used to correct the measured particle intensity for the local background at each time point.

## Results

### Single HIV-1 fusion visualized by viral content release

**Fig 1:**
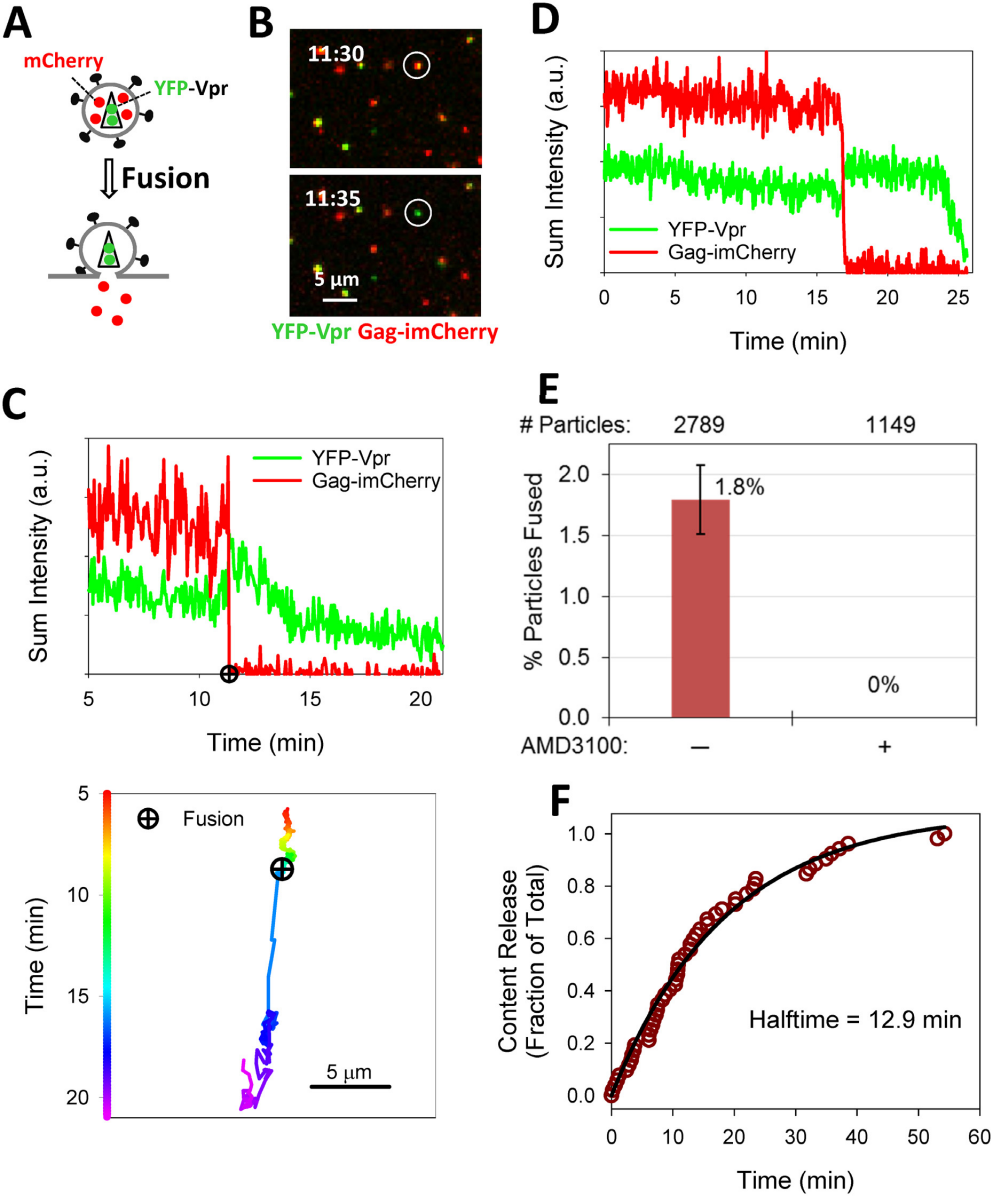
Detection of single HIV-1 fusion based on release of the mCherry viral content marker. (A) Illustration of viral fusion detection with HXB2 Env pseudotyped particle (HXB2pp) co-labeled with releasable content marker Gag-imCherry (red) and core-associated marker YFP-Vpr (green). (B) HXB2 pseudovirus co-labeled with Gag-imCherry (red) and YFP-Vpr (green) fuses with CV1/CD4/CXCR4 cell as detected by instantaneous loss of mCherry signal. (C) Fluorescence intensity profiles (top) and the virus trajectory (bottom) obtained by tracking the fusing particle in panel B. (D) Fluorescence intensity profiles of another fusing HXB2pp particle. (E) Efficiency of HXB2 Env-mediated single particle fusion in CV1/CD4/CXCR4 cells untreated and treated with 10 μM AMD3100. Total number of particles examined for each condition listed at top of graph. (F) Kinetics of single HXB2pp fusion shown as a cumulative plot of the fraction of fused particles over time. Solid line is obtained by single exponential curve fitting.

We have recently implemented a reliable assay for visualization of single virus fusion [24, 27, 32]. Instead of co-labeling pseudoviruses with content and membrane markers, as described previously [3, 4], we labeled viruses with a fiducial core-incorporated fluorescent protein, and a releasable content marker. The HIV-1 Vpr tagged with a fluorescent protein (originally introduced in [33]) incorporated into the viral core provides a reference signal for single particle tracking before and after the fusion. Viral fusion is detected by loss of the content marker Gag-imCherry (Fig 1A). In Gag-imCherry, the fluorescent protein is inserted between the matrix and capsid domains of Gag, flanked by the protease cleavage sites, as described in [4, 27, 34]. Cleavage of Gag-imCherry upon virus maturation produces free mCherry, which is readily released upon virus fusion [27]. Furthermore, tagging Vpr with the pH-sensitive YFP protein (YFP-Vpr) affords a means to detect small fusion pores, independent of the release of viral content, based upon changes in the intraviral pH [27].

As shown previously [27, 32], fusion of YFP-Vpr/Gag-imCherry co-labeled particles pseudotyped with HXB2 Env (HXB2pp) results in the loss of mCherry, whereas the YFP-Vpr signal remains detectable (Fig 1B). Moreover, coincident with the pore formation (release of mCherry), the YFP-Vpr signal exhibits transient increase, likely due to the increase in the intraviral pH as a result of exposure to the cytoplasm. In agreement with our previous study [32], the YFP-Vpr signal tended to decay over the course of several minutes (Fig 1C and 1D), apparently due to dissociation of this marker from the viral core. The virus trajectory (Fig 1C) is consistent with viral fusion occurring with an early endosome, before the particle undergoes directional microtubule-mediated transport, which is linked to endosome maturation and acidification of its lumen [35–38]. These single virus imaging experiments enable the measurements of the extent and kinetics of HIV-1 fusion with target cells. Consistent with our published results [3], only 1.8% of double-labeled HXB2pp underwent fusion with CV-1-derived target cells (Fig 1E) and the rate of fusion was relatively fast (half-time ~10 min, Fig 1F). In control experiments, HXB2pp content release was abrogated by the CXCR4 antagonist, AMD3100 (Fig 1E) and by the gp41 refolding inhibitor C52L (data not shown). These results further demonstrate that single HIV-1 fusion can be reliably detected based upon mCherry release. However, this labeling approach does not generally discriminate between virus fusion with the plasma membrane and fusion with endosomes. Fusion at either entry site should result in loss of mCherry and retention of YFP-Vpr signal.

**Fig 2:**
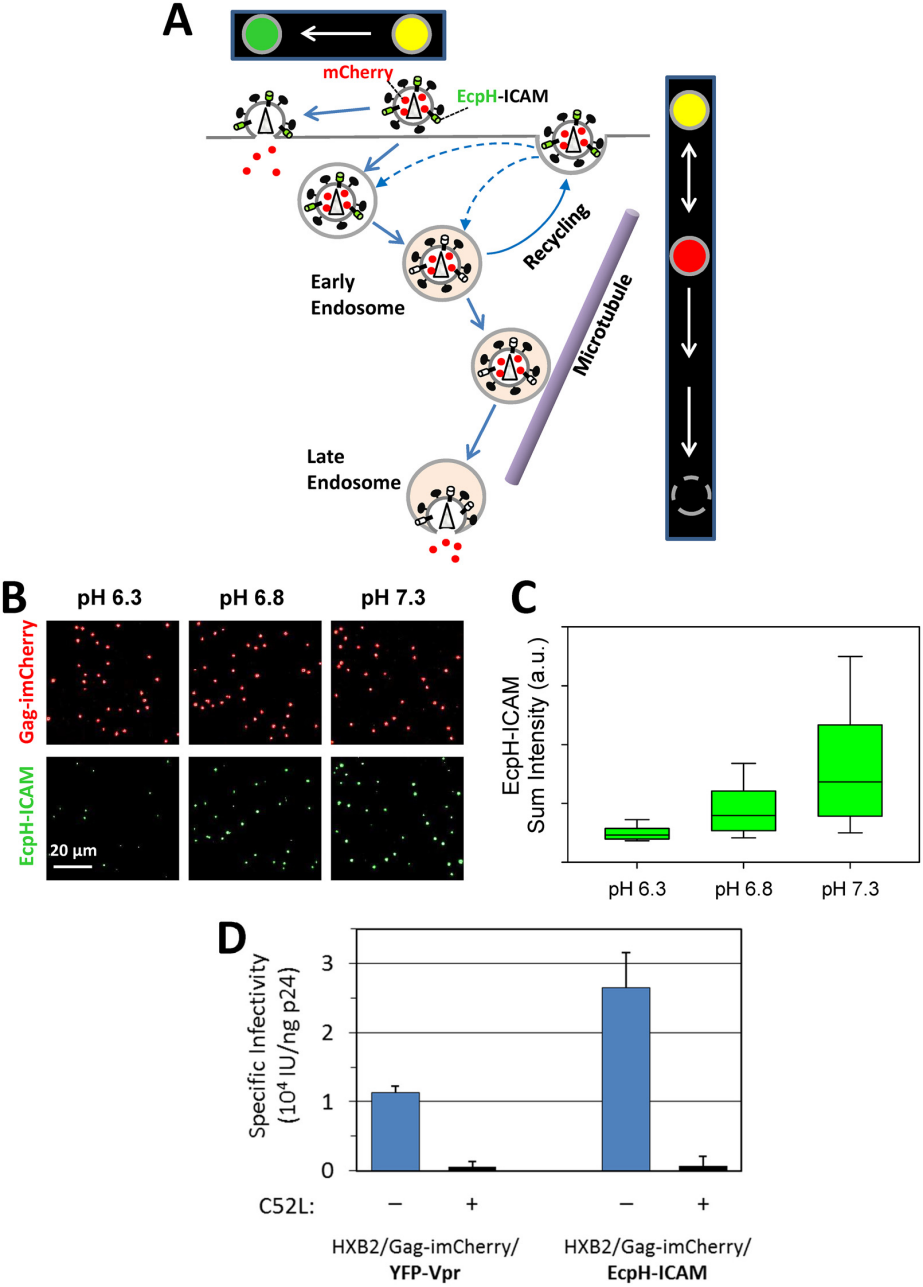
Strategy to detect rare viral fusion events at the cell surface. (A) Illustration of fusion, recycling and retrograde trafficking of viral particles co-labeled with EcpH-ICAM (pH-sensitive membrane marker, green) and Gag-imCherry (releasable content marker, red). Changes in particle’s fluorescence signal (in false colors) associated with fusion at the plasma membrane and entry/fusion with acidic endosomes is shown in boxes above and on the right of the cartoon. Due to the loss of EcpH reference signal before fusion with acidic endosomes, these events are not detected. (B) HXB2pp co-labeled with Gag-imCherry (red) and extra-viral pH sensor EcpH-ICAM (green) were bound to poly-L-lysine coated coverslips and imaged at room temperature in buffers adjusted to pH 6.3, 6.8 or 7.3. (C) Plot shows the distribution of EcpH-ICAM background-subtracted sum intensity of more than 3,000 virus particles at each pH. Green bar shows median and interquartile range and whiskers contain the middle 80% of the intensity distribution. (D) Comparison of specific infectivity (infectious units per nanogram p24; IU/ng p24) of HXB2pp containing Gag-imCherry and YFP-Vpr or EcpH-ICAM untreated and treated with 5 μM C52L.

In order to test whether HIV-1 can fuse with the plasma membrane we incorporated the EcpH-ICAM chimera [15, 31], comprised of the pH-sensitive fluorescent protein ecliptic pHlourin (EcpH [39]) linked to the transmembrane domain fragment of ICAM-1, into the membrane of HXB2pp. Because ICAM-1 can incorporate into HIV-1 [40–44], it was chosen as an anchor for the extra-viral pH sensor. The pseudoviruses were co-labeled with the releasable viral content marker, Gag-imCherry. Since EcpH-ICAM is nearly fully quenched at mildly acidic pH ~6.3 ([31] and Fig 2A–2C), its use as a fiducial marker for tracking viral content release disregards fusion events that occur with acidic endosomes (Fig 2A). By contrast, HIV-1 fusion occurring at neutral pH, either at the plasma membrane or in early pH-neutral endosomes, should be readily detectable, using the EcpH-ICAM signal to track particles that lose the mCherry marker. Here we assume that the 2-dimensional lateral diffusion of the ICAM-based probe is slower than the release (3D diffusion) of the small content marker through a fusion pore, which is completed much faster than we can resolve [27]. We have previously used a similar virus labeling strategy to measure trafficking into acidic compartments, but the deliberate usage of the C-terminally tagged Gag, which is not cleaved by the viral protease, precluded the detection of fusion events [31]. Of note, the infectious titers of particles labeled with EcpH-ICAM/imCherry and those labeled with YFP-Vpr/Gag-imCherry were similar (Fig 2D), suggesting that the incorporation of an extra-viral pH-sensor does not adversely affect the virus’ infectivity.

### Quenching of viral EcpH-ICAM fluorescence reports HIV-1 entry into acidic endosomes

**Fig 3:**
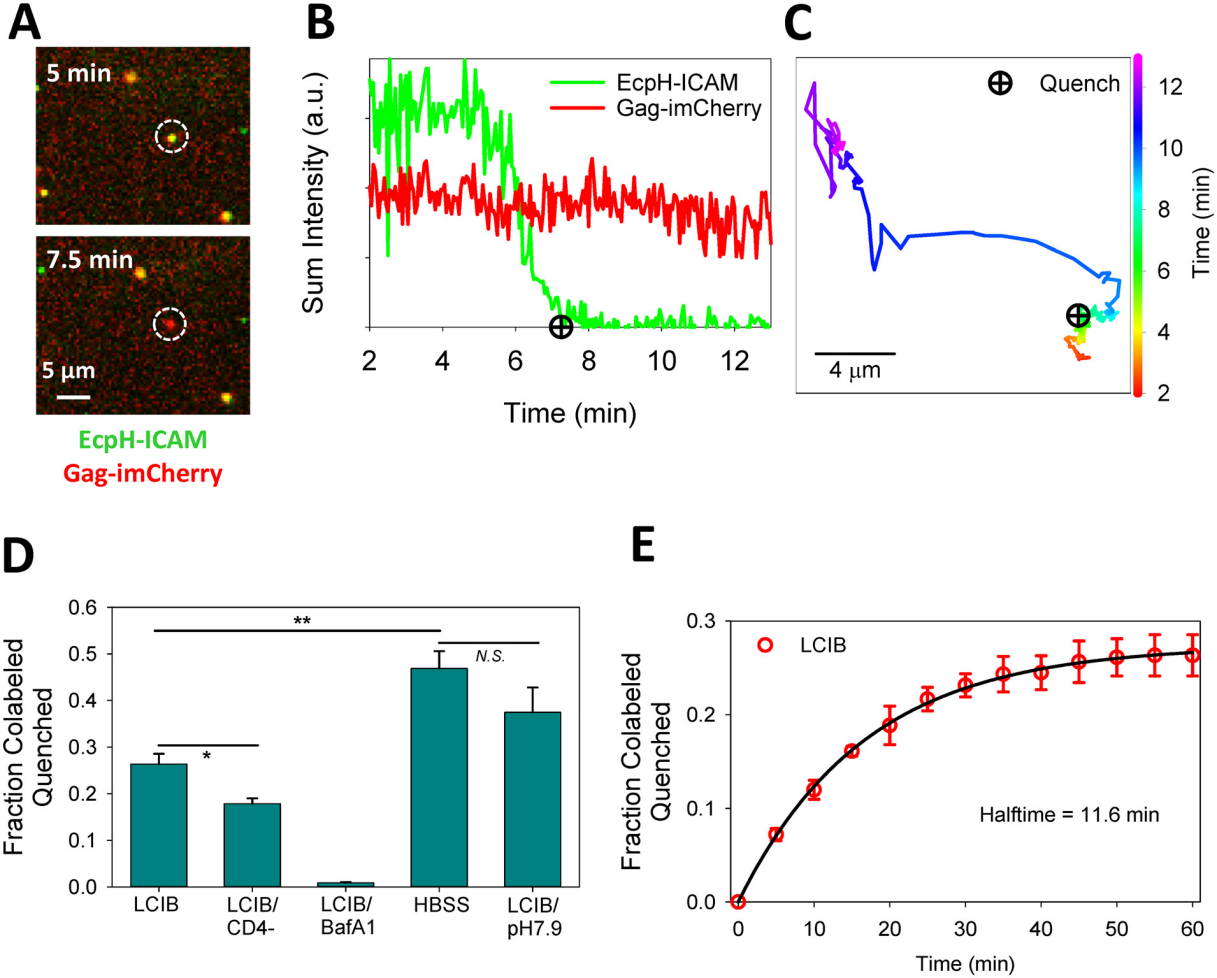
Single particle analyses of HIV-1 entry into acidic endosomes. (A) HXB2pp co-labeled with EcpH-ICAM and Gag-imCherry enters into a mildly acidic compartment of a CV1/CD4/CXCR4 cell, as detected by quenching of EcpH-ICAM signal. (B, C) Fluorescence intensity profiles and trajectory obtained from tracking the virus particle in panel A show quenching of EcpH signal and subsequent directional movement of the particle. Quenching of EcpH prior to directional movement of the particle is consistent with the initial acidification to pH 6.0 typical for early endosomes. (D) Extent of single-virus particle quenching in CV1/CD4/CXCR4 cells shown as fraction of total particles quenched in one hour experiment for the following conditions: in HBSS, LCIB with or without 100 nM Bafilomycin A1 (BafA1) or LCIB adjusted to pH 7.9. The extent of EcpH-ICAM quenching in parental CV1 cells in LCIB (LCIB/CD4-) is also shown. Error bars are standard error of the mean for three independent experiments. *p<0.05, **p<0.01, “N.S.”p>0.05, student’s t-test. (E) Kinetics of EcpH-ICAM quenching in CV1/CD4/CXCR4 cells bathed in LCIB. Symbols and error bars (red) are mean and standard error of the mean for three independent experiments and curve (black) is fit to single-exponential model.

Entry of EcpH-ICAM/Gag-imCherry labeled HXB2pp into acidic intracellular compartments was manifested by the loss of EcpH fluorescence. Single particle tracking revealed that, as observed previously [15, 31], the EcpH signal from individual particles was fully quenched over the period of about one minute (Fig 3A–3C), most likely due to gradual acidification of endosomal lumen. The majority of particles did not lose mCherry following the EcpH quenching events, i.e. did not fuse, and their endosomal transport could be visualized for considerable time periods (e.g. Fig 3A–3C). The kinetics and extent of single HXB2pp entry into acidic endosomes (complete quenching of EcpH-ICAM) are shown in Fig 3D and 3E. HIV-1 uptake was significantly (p = 0.03) promoted by interactions with cellular receptors. Whereas nearly 30% of double-labeled HXB2pp entered into acidic compartments of CV-1 cells expressing CD4 and CXCR4 within the first hour of incubation at 37°C, only ~15% were internalized by parental CV-1 cells lacking these receptors (Fig 3D). As expected, treatment with BafA1 to raise endosomal pH abrogated EcpH quenching.

About 30% of HXB2pp was internalized by CV-1-derived cells within one hour at 37°C (Fig 3D and 3F). This is in contrast to uptake by HeLa-derived target cells where nearly all HXB2 particles internalize or detach in the same time interval [31]. We found that particles adhered to endocytically inactive cells remained relatively immobile and were not internalized by the end of the experiment. The extent and kinetics of EcpH quenching (Fig 3E) define the time window for detecting viral fusion at neutral pH.

The above HXB2pp uptake experiments were done in Live Cell Imaging Buffer (LCIB, Invitrogen). Since we previously performed single HIV-1 fusion experiments in Hank’s buffer (HBSS) [4, 31], we asked whether the choice of buffer might affect the efficiency of virus uptake. Surprisingly, cells bathed in HBSS more avidly internalized HXB2pp than those bathed in LCIB (p = 0.009). Approximately 45% of double-labeled viruses lost their EcpH signal within the first hour of incubation with cells (Fig 3D). Unlike the HEPES-buffered LCIB, HBSS is a CO_2_-dependent buffer which reaches pH around 8.0 under ambient conditions. We therefore asked whether the difference in the virus uptake/acidification rates was due to the alkaline extracellular pH. Indeed, when imaging experiments were done in LCIB adjusted to pH 7.9, the extent of EcpH quenching at the end of 1 h was close to that in HBSS (Fig 3D). These results indicate that the extent of HIV-1 internalization and/or acidification of early endosomes carrying the virus is affected by extracellular pH. In order to maximize the chances of detecting HIV-1 fusion at neutral pH, we primarily used LCIB (pH 7.3), in which less than 30% of particles lost the EcpH signal (Fig 3D) in one hour, throughout this study.

### Internalized HIV-1 can be recycled to the cells surface

**Fig 4:**
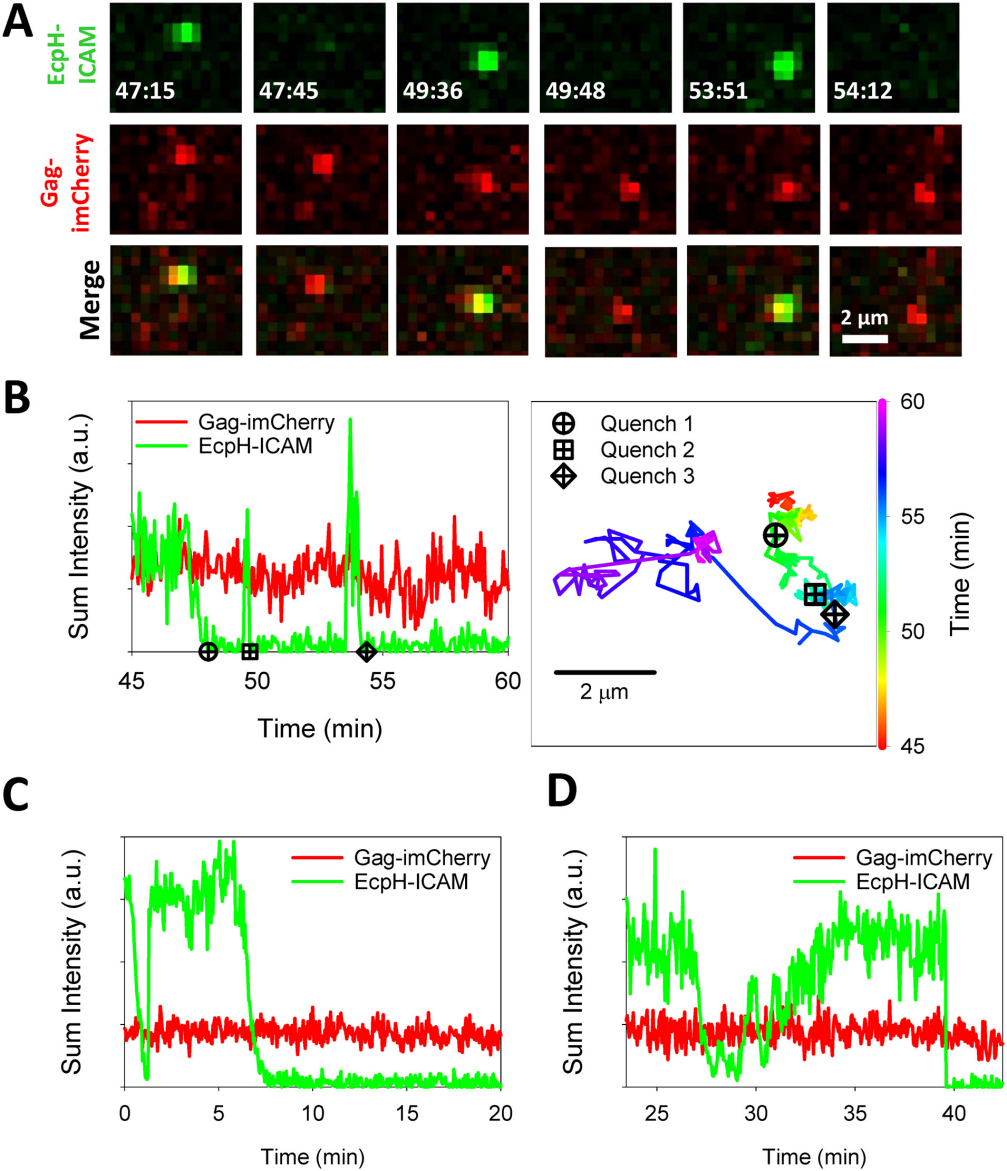
Blinking of viral EcpH-ICAM marker during HXB2 pseudoparticle entry into cells. (A) HXB2pp co-labeled with releasable content marker Gag-imCherry (red) and extra-viral pH sensor EcpH-ICAM (green) shuttles between neutral and acidic compartments of CV-1/CD4/CXCR4 cells, as detected by cycles of quenching and subsequent dequenching (blinking) of EcpH-ICAM signal. (B) Fluorescence intensity profiles and the trajectory of particle shown in panel A. (C, D) Additional examples of EcpH blinking upon HXB2pp entry into cells. See also S1 Movie.

After losing the EcpH-ICAM signal, a fraction of double-labeled particles exhibited reversible EcpH dequenching/quenching, termed “blinking” (Fig 4A and 4B and S1 Movie). During blinking, the EcpH signal switched between fluorescent and fully quenched states. A range of blinking behaviors was observed—from multiple dequenching events for a given particle, to a prolonged dequenched state (Fig 4B–4D). In either case, at later times the EcpH signal was eventually permanently lost, while the mCherry signal persisted. BafA1 was found to abrogate the blinking behavior, by eliminating EcpH quenching (data not shown). Switching of EcpH-ICAM signal between fluorescent and non-fluorescent states strongly implies that viruses entering acidic compartments are recycled to the cell surface. This notion is supported by the limited motility of the blinking particles, as exemplified by the particle tracked in Fig 4B, which exhibits directional movement only after the blinking activity has ceased. Importantly, we did not observe the mCherry content release for particles exhibiting the blinking behavior.

**Fig 5:**
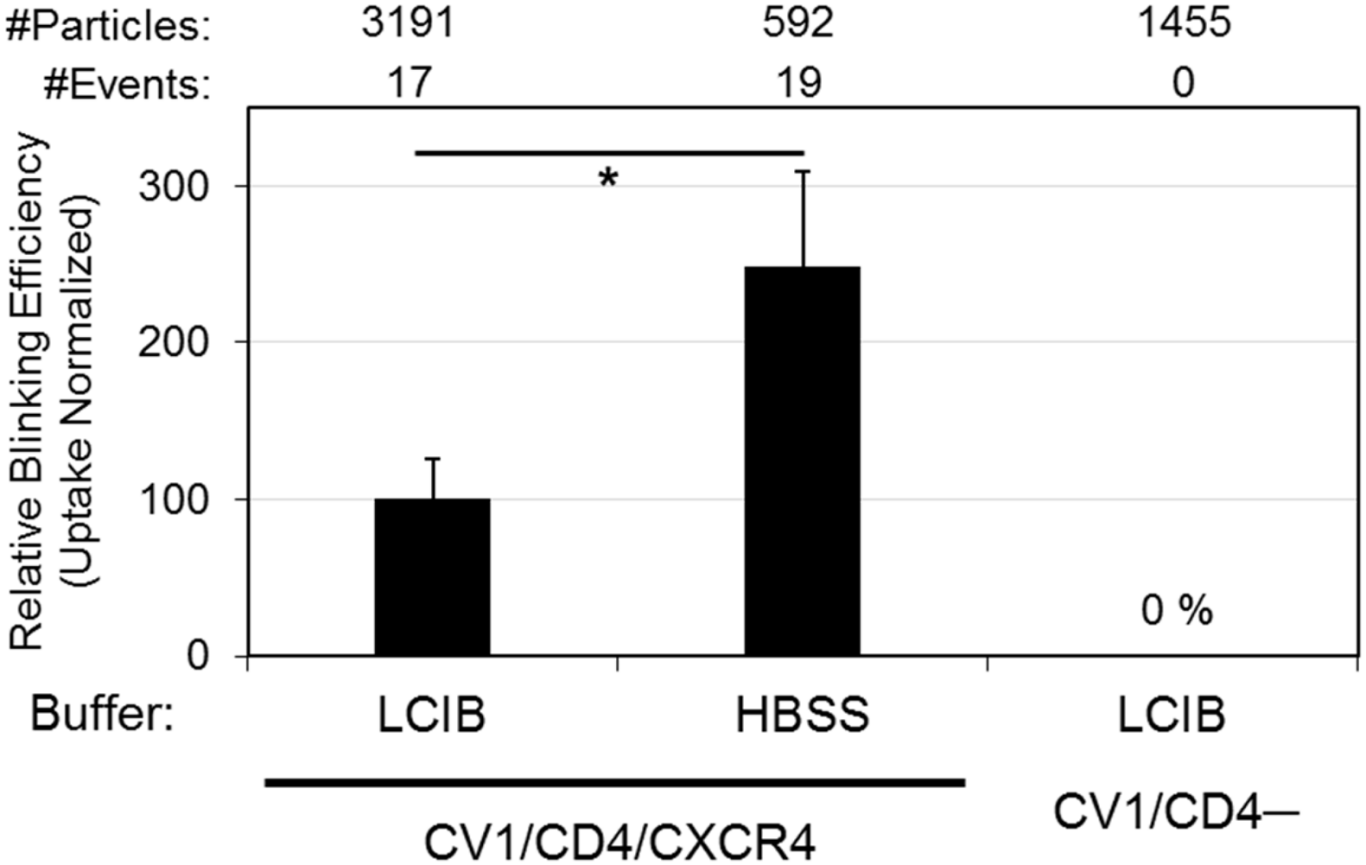
EcpH-ICAM blinking efficiency under different conditions. HXB2 pseudoparticles co-labeled with Gag-imCherry and EcpH-ICAM were pre-bound to cells and imaged at 37°C for 60 min. Blinking events were detected as quenching/dequenching of EcpH-ICAM label. Plot shows the EcpH blinking efficiency measured in LCIB and HBSS in CV1/CD4/CXCR4 cells and in LCIB-bathed parental CV-1 cells lacking CD4. Total number of particles examined for each condition listed at top of plot. The fraction of particles exhibiting blinking behavior was normalized to the fraction of particles entering into acidic compartments, as evidenced by permanent EcpH-ICAM quenching (Fig 3D). *p<0.05, student’s t-test.

While the relatively slow loss of EcpH fluorescence over ~1 min during the first quenching event likely reflects a gradual drop of the pH in endosomal lumen, fast recovery of green signal appears to represent particle release to the cell surface through a one-step exocytic event. However, rare particles exhibited slow recovery of EcpH fluorescence (Fig 4D). The mechanism for such a complex fluorescence profile remains to be elucidated. The observed EcpH blinking occurred as a result of HIV-1 interactions with cognate receptors, since EcpH dequenching was never detected in CV-1 cells lacking CD4 (Fig 5). Interestingly, more EcpH blinking was observed in HBSS compared to LCIB solution. The effect of HBSS on blinking thus appears to correlate with the promotion of virus uptake (Fig 3D). These results indicate that HIV-1 can traffic through different intracellular routes after engaging receptor/co-receptor.

### HIV-1 particles co-labeled with EcpH-ICAM and Gag-imCherry can release content through spontaneously formed membrane defects

**Fig 6:**
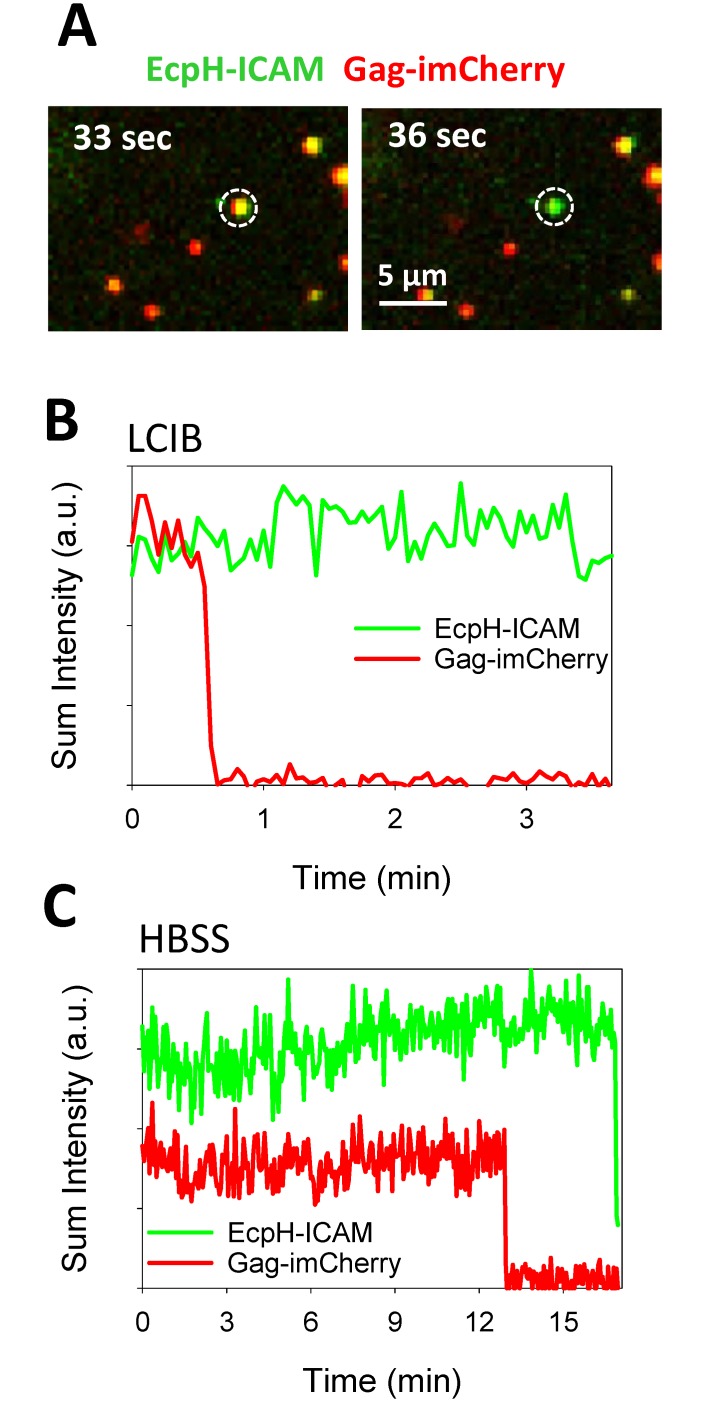
Detection of viral content release at neutral pH. (A) Snapshot of HXB2pp (white circle) co-labeled with Gag-imCherry (red) and EcpH-ICAM (green) before and after release of mCherry content marker in interaction with CV1/CD4/CXCR4 cell in LCIB. (B) Fluorescence intensity profiles of the particle shown in panel A (in LCIB). (C) Fluorescence intensity profiles of HXB2pp content release event at neutral pH on cells bathed in HBSS. See also S2 Movie.

**Fig 7:**
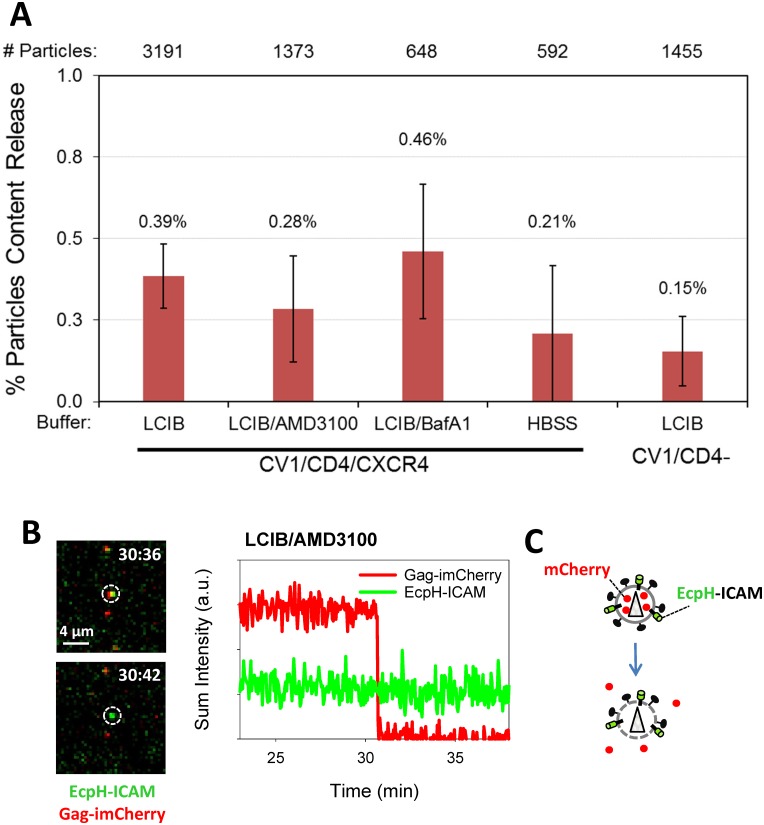
Efficiency of HIV-1 content release at neutral pH. HXB2pp labeled with releasable content marker Gag-imCherry and extra-viral pH sensor EcpH-ICAM were pre-bound in the cold to cells and imaged at 37°C for 60 min. (A) Graph shows the content release efficiency measured in LCIB, LCIB supplemented with 100 nM BafA1 or HBSS with CV1/CD4/CXCR4 cells, as well as with CV-1 cells lacking CD4 (CV1/CD4-) bathed in LCIB. In control experiments, fusion was carried out in the presence of 10 μM AMD3100. Total number of particles examined for each condition listed on top of plot. Statistical analyses performed using two tests (nonparametric Mann-Whitney test and zero-inflated Poisson model) did not reveal significant differences between either of the conditions tested. (B) Snapshot of HXB2pp (white circle) before and after content release in interaction with CV1/CD4/CXCR4 cell during treatment with 10 μM AMD3100 and corresponding fluorescent intensity profile of particle. (C) Illustration of loss of mCherry through defects in the viral membrane.

To determine whether HIV-1 can fuse with the plasma membrane, we imaged single HXB2pp co-labeled with EcpH-ICAM and Gag-imCherry. As above, a fraction of particles lost the EcpH signal due to entry into acidic compartments, which precluded reliable detection of subsequent fusion events. However, a very small fraction of double-labeled pseudoviruses released the mCherry content marker while retaining the EcpH signal (Fig 6A and 6B and S2 Movie). For those rare events, the steady level of EcpH fluorescence shows that viral content release occurred at neutral pH—either on the cell surface or in early pH-neutral vesicles. These events were observed regardless the choice of an extracellular buffer (LCIB vs. HBSS, Fig 6A–6C). Similar fractions of double-labeled particles bound to CV1-derived cells lost mCherry at neutral pH in LCIB and HBSS (0.39% and 0.21%, respectively, Fig 7A).

The viral content release at neutral pH should be more readily observed in the presence of BafA1 which blocks endosomal acidification, thereby extending the time window for detecting HIV-1 fusion at neutral pH. However, the marginal increase in the extent of mCherry release at neutral pH in LCIB supplemented with BafA1 compared to untreated cells was not statistically significant (Fig 7A). If the fusion competence of EcpH-ICAM labeled virus is comparable to that of YFP-Vpr labeled virus (Fig 1E), one would expect to see a more substantial enhancement of mCherry release events in the presence of BafA1, perhaps approaching the optimal level of fusion which is ~2% of co-labeled particles (Fig 1 and [3]). The lack of stronger enhancement of content release from EcpH-ICAM/Gag-imCherry labeled viruses (Fig 7A) could be indicative of their relatively poor fusogenic activity. However, the higher specific infectivity of this preparation compared to HXB2pp co-labeled with YFP-Vpr and Gag-imCherry (Fig 2D) argues against this possibility. To conclude, only a low level of mCherry release events could be detected for HXB2pp co-labeled with EcpH-ICAM and Gag-imCherry on CV-1 derived cells under the experimental conditions employed in this work.

We then asked whether the observed release of mCherry from EcpH-ICAM labeled particles was due to the HXB2 Env-mediated viral fusion. Unexpectedly, we did not observe a significant reduction in the probability of mCherry release at neutral pH in the presence of a high dose of the HXB2 fusion inhibitor AMD3100 (Fig 7A and 7B). At the concentration used in these experiments, AMD3100 abrogated fusion of HXB2pp co-labeled with YFP-Vpr and Gag-imCherry (Fig 1E). The inability of AMD3100 to block mCherry release from HXB2pp implies that the content marker is lost to the extracellular solution through spontaneous formation of a viral membrane defect and not through a fusion pore connecting the virus interior with the cytoplasm. The instantaneous and complete loss of the mCherry signal from single particles indicates that lytic events are occurring on the cell surface, since content release into early pH-neutral endosomal vesicles should not lead to immediate disappearance of fluorescent signal. The fact that viral content release could also be detected in control experiments with parental CV-1 cells lacking CD4 (Fig 7A) further supports the membrane defect rather than fusion-related mechanism of mCherry loss at the cell surface.

**Fig 8:**
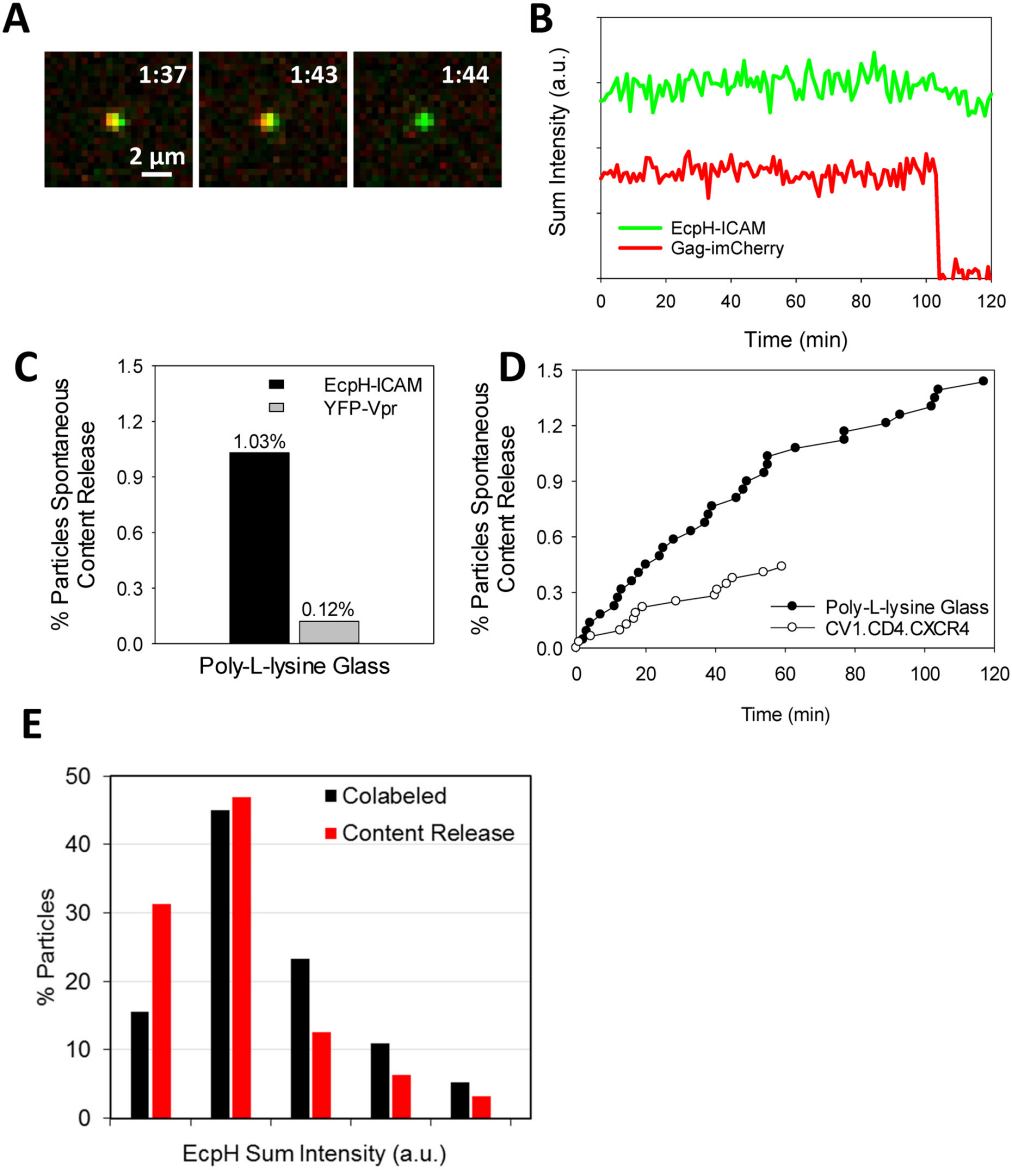
Characterization of spontaneous content release from labeled HXB2pp adhered to coverslips. (A) Images of spontaneous content release from a single HXB2pp co-labeled with Gag-imCherry/EcpH-ICAM. Pseudoviruses were attached to poly-L-lysine coated glass coverslips in the cold and imaged in LCIB at 37°C. (B) Fluorescence intensity profiles of particle shown in panel A. (C) Comparison of content release efficiency from immobilized HXB2pp co-labeled with Gag-imCherry/EcpH-ICAM and Gag-imCherry/YFP-Vpr during 60 min image acquisition. (D) Kinetics of single content release events on poly-L-lysine coated glass and on CV1/CD4/CXCR4 cells shown as cumulative of events per total particles over time (symbols). (E) Distribution of immobilized HXB2pp on poly-L-lysine coated glass with background-subtracted EcpH intensity for all co-labeled particles (black) and co-labeled particles that spontaneously lyse (red). See also S3 Movie.

To test the notion that the observed rare mCherry release events occur independently of Env-receptor interactions, we examined release of mCherry from pseudoviruses immobilized on poly-lysine coated coverslips. Occasional spontaneous mCherry loss from immobilized HXB2pp co-labeled with EcpH-ICAM was observed: ~1% of double-labeled particles released content within 1 h-incubation at 37°C in LCIB (Fig 8A–8C and S3 Movie). In fact, the rate of lysis was ~2-fold greater on coverslips compared to CD4-expressing cells (Fig 8D), suggesting that adhesion to poly-lysine coated glass may somehow destabilize the particles. While the factors causing mCherry release through a defect in the HXB2pp membrane are not known, it is notable that immobilized particles co-labeled with YFP-Vpr and Gag-imCherry were much less prone to spontaneous release of mCherry than those co-labeled with EcpH-ICAM and Gag-imCherry (Fig 8C). This appears to indicate that virus instability might be caused by virus-incorporated ICAM-1-derived proteins. However, we did not detect a clear correlation between the amount of EcpH-ICAM incorporated into HXB2pp and release of mCherry (Fig 8E).

Collectively, the above results imply that incorporation of EcpH-ICAM into HXB2pp may destabilize the viral membrane, resulting in rare spontaneous release of a small viral content marker. This release precludes reliable detection of viral fusion at neutral pH, even in the presence of BafA1. Thus, whereas the sensing of extraviral pH should provide a means to pinpoint the sites of HIV-1 fusion, a combination of EcpH-ICAM and Gag-imCherry probes appears to interfere with the fusion assay by adding a background of spontaneous mCherry release events.

## Discussion

In this study, we have devised a single virus imaging-based approach to sensitively and selectively detect single HIV-1 fusion events at the cell surface or in early pH-neutral endosomes. This was accomplished by co-labeling pseudoviruses with a pH-sensor, which is quenched under mildly acidic conditions, and a releasable content marker. Surprisingly, a small fraction (~0.4%) of EcpH-ICAM/Gag-imCherry, but not YFP-Vpr/Gag-imCherry labeled particles, released mCherry at neutral pH through a mechanism unrelated to HIV-1 Env-mediated fusion. Content release from EcpH-ICAM labeled viruses occurred with similar probability on the surface of receptor-expressing and control cells lacking CD4, regardless of the presence of HIV-1 fusion inhibitors, and even on coverslips. It thus appears that most, if not all, content release events from EcpH-ICAM viruses on target cells are due to spontaneous membrane defect formation. We cannot rule out the possibility that the diffusion of EcpH-ICAM into the plasma membrane as a result of fusion occurs quicker than we could resolve. While we consider this an unlikely scenario, simultaneous loss of both membrane and content markers would not allow the discrimination between fusion and virus detachment from the plasma membrane, as documented previously [31, 45].

Currently, the mechanism of the rare mCherry release events involving HIV-1 pseudoviruses is unclear. Since the particles co-labeled with YFP-Vpr and Gag-imCherry were approximately 10-fold more resistant to spontaneous mCherry release than particles co-labeled with EcpH-ICAM and Gag-imCherry, it appears that incorporation of EcpH-ICAM into virions could be, at least partially, responsible for destabilization of viral particles. However, we did not observe a clear correlation between the amount of EcpH-ICAM and the propensity of viruses to lyse (Fig 8E). Moreover, pseudoviruses containing the full-length ICAM-1 exhibited only a 2-fold increase in the extent of spontaneous content release (data not shown). Regardless of the causes for viral content release through membrane defects, our results show that caution should be exercised when interpreting the loss of a small content marker observed in imaging experiments.

Another unexpected observation was that BafA1 treatment did not markedly increase the extent of fusion of EcpH-ICAM/Gag-imCherry labeled particles (Fig 7A). Under these conditions, the EcpH signal should remain unquenched, thus allowing particle tracking and visualization of fusion in endosomal compartments. The lower likelihood of content release from EcpH-ICAM/Gag-imCherry particles in the presence of BafA1 as compared to YFP-Vpr/Gag-imCherry virions in the absence of this drug is in contrast to the greater specific infectivity of the former virus (Fig 2D). It is unclear whether particles that spontaneously release mCherry remain fusion competent or capable of establishing productive infection. It is possible that membrane defects through which the content marker is released are small and/or transient and that the overall integrity and infectivity of a virion is preserved. This notion appears to be supported by the relatively high specific infectivity of EcpH-ICAM/Gag-imCherry pseudoviruses.

ICAM-1-derived viral membrane marker has been widely used by our group to study virus uptake and fusion with acidic endosomes [15, 27, 31, 37, 38, 46]. However, all previous studies used EcpH-ICAM or other ICAM-1-derived pH-sensors either in the context of pseudoviruses based on the MLV Gag or HIV-1 pseudoviruses labeled with a non-releasable Gag-mCherry, in which the fluorescent protein was fused to the C-terminus of Gag. It therefore appears that imaging single HIV-1 particles co-labeled with EcpH-ICAM and HIV-1 Gag-imCherry reveals the previously unappreciated instability of the viral membrane that is manifested in the loss of content from a small fraction of particles.

In addition to reporting the virus entry into acidic endosomes, the EcpH-ICAM-based pH sensor revealed that internalized particles can be recycled to the cell surface and re-internalized. It is unclear whether HIV-1 recycling plays a role in the productive entry process. We cannot rule out the possibility that blinking particles fuse after permanently re-entering into acidic endosomes. However, rare particles that exhibited the “blinking” behavior did not release their content while transiently residing at the plasma membrane before the re-internalization step. Regardless of the functional relevance of this finding, our imaging assay demonstrates that HIV-1 is internalized through alternative endocytic pathways and provides a tool to study trafficking and other viruses. Incorporating a pH-insensitive viral core marker into particles co-labeled with EcpH-ICAM and HIV-1 Gag-imCherry will enable continuous tracking of viral uptake and fusion in acidic compartments by 3-color live cell microscopy. The utility of this and similar approaches is supported by our recent study, in which an ICAM-based pH sensor consisting of a fluorescence donor-acceptor pair has been incorporated into MLV-based particles pseudotyped with Avian Sarcoma and Leukosis Virus Env glycoprotein [38]. This labeling strategy afforded a unique opportunity to measure the pH changes around internalized viruses and simultaneously visualize viral fusion with acidic endosomes. The adverse effect of ICAM-derived probes on the integrity of the viral membrane highlights the need to explore additional candidate proteins to use as the viral membrane anchor for the pH sensor, such as those naturally enriched in HIV-1 membranes [47].

## Supporting Information

S1 Movie Extra-viral pH-sensor blinks as HIV-1 pseudoparticle shuttles between neutral and acidic cellular compartments. HXB2 pseudoparticles labeled with releasable content marker Gag-imCherry (red) and extra-viral pH sensor EcpH-ICAM (green) were pre-bound in the cold to CV1/CD4/CXCR4 cells and imaged for 1 hour at 37°C. Particle trafficking to mildly acidic cellular compartment completely quenches fluorescence from the EcpH-ICAM label. ~0.8% of particles exhibited dequenching of EcpH-ICAM following quenching, suggesting trafficking of the particle back to a neutral compartment or the cell surface.
(AVI)

S2 Movie HIV-1 pseudoparticle releases viral content at neutral pH. HXB2 pseudoparticles labeled with releasable content marker Gag-imCherry (red) and extra-viral pH sensor EcpH-ICAM (green) were pre-bound in the cold to CV1/CD4/CXCR4 cells and imaged for 1 hour at 37°C. Approximately 0.4% of the pseudoparticles released viral content before the quenching of the EcpH-ICAM label due to trafficking to a mildly acidic cellular compartment.
(AVI)

S3 Movie HIV-1 pseudoparticle immobilized on glass spontaneously releases viral content. HXB2 pseudoparticles labeled with releasable content marker Gag-imCherry (red) and extra-viral pH sensor EcpH-ICAM (green) were pre-bound in the cold to poly-L-lysine coated cover glass and imaged at 37°C for 1 hour. ~1% of the pseudoparticles spontaneously released viral content.
(AVI)
